# The Gut Microbial Adaptation of Wild Goitered Gazelles Under Antibiotic Pressure in the Qaidam Basin

**DOI:** 10.3390/microorganisms13081842

**Published:** 2025-08-07

**Authors:** Qing Zhao, Yiran Wang, Jingqing Ma, Wen Qin

**Affiliations:** 1 State Key Laboratory of Plateau Ecology and Agriculture, Qinghai University, Xining 810016, China; zhaoqing1063@163.com (Q.Z.); w18609783019@126.com (Y.W.); 2 School of Ecological and Environmental Engineering, Qinghai University, Xining 810016, China; 13997128125@163.com

**Keywords:** goitered gazelles, gut microbiota, antibiotic pressure

## Abstract

Gut microbiota plays a vital role in host resilience but may be disrupted under environmental antibiotic pressure. The goitered gazelle (*Gazella subgutturosa*), a keystone ungulate in the Qaidam Basin, is crucial for ecosystem stability. We aimed to investigate how this species responds to antibiotic pressure through gut microbial adaptation. Using 16S rRNA sequencing and weighted gene co-expression network analysis (WGCNA) on fecal and soil samples from six regions, we identified 18 microbial modules, of which three were strongly associated with antibiotics (|r| ≥ 0.75, *p* < 0.05). Gut microbial α-diversity was lowest in the antibiotic-rich, vegetation-poor TGL region and highest in XRH, where diverse vegetation appeared to buffer antibiotic impact. Antibiotic pressure can reshape gut microbial communities, exerting both adaptive benefits and adverse effects. High-quality habitats may alleviate the negative impacts of antibiotic pressure.

## 1. Introduction

The gut microbiota plays a vital role in host adaptation by co-evolving with the host and mediating physiological responses to environmental stressors, including antibiotic residues [1,2]. Growing evidence indicates that gut microbial communities in wild animals are both impacted by and responsive to antibiotic exposure. Such exposure can reduce microbial diversity and induce dysbiosis [3]. While certain taxa contribute to host resilience via metabolic detoxification and maintenance of intestinal homeostasis, this is the adaptive shift of gut microbiota [4]. Wild ungulates, due to their wide distribution and mobility across human-influenced landscapes, serve as effective bioindicators for tracking antibiotic contamination [5,6]. Therefore, understanding gut microbiota–antibiotic interactions in wild herbivores is critical for assessing ecological risks and informing conservation and environmental management strategies [7].

Antibiotics constitute a widespread environmental stressor capable of altering gut microbiota composition in wild vertebrates. Even low, environmentally relevant concentrations reduce gut microbial α-diversity and functional redundancy, potentially compromising host health and ecological resilience [8,9]. Empirical studies support these effects: Free-ranging sika deer from contaminated rangelands show enrichment of glycoside hydrolases and carbohydrate-binding modules in the CAZy repertoire compared to captive individuals [10]. Likewise, antibiotic administration in horses reduces Firmicutes (notably *Clostridia*) and increases Bacteroidetes and *Prevotella*, resulting in dysbiosis and impaired host function [11]. These findings highlight the destabilizing impact of chronic antibiotic exposure on gut microbial ecology in wild herbivores.

The goitered gazelle (*Gazella subgutturosa*) [12] inhabits arid and semi-arid environments, including flat grasslands, interdune depressions, and dwarf-shrub steppe within alpine-desert zones—areas overlapping with potential antibiotic exposure. As the only wild ungulate widely distributed across the core habitat of Qaidam Basin, it occupies diverse microhabitats and serves as a sentinel for regional antibiotic pressure. As a dominant herbivore, it plays a keystone role in maintaining food web stability and ecosystem function [13,14]. This species exhibits gut microbial plasticity in response to environmental stress. Seasonal metagenomic data reveal winter increases in Firmicutes (notably *Christensenellaceae*) and Bacteroides, alongside enriched energy-metabolism pathways, supporting enhanced nutrient extraction [15]. Its gut microbiota also harbors diverse antibiotic resistance genes (unpublished), indicating environmental acquisition and dissemination potential. Despite its ecological importance, *G. subgutturosa* faces intensified pressures from habitat fragmentation, livestock competition, and climate change, contributing to a population decline to fewer than 49,000 individuals; it is currently listed as Vulnerable on the IUCN Red List [16]. However, microbiome-mediated adaptations to antibiotic exposure remain poorly understood [13,17,18].

This study will (i) quantify antibiotic residues across key habitats of the Qaidam Basin and (ii) assess their association with gut microbiota diversity shifts in the goitered gazelle. By coupling environmental residue profiling with gut microbiome, we aim to determine whether gut microbial reassemblies constitute a core adaptive mechanism that enables goitered gazelles to persist under chronic, multi-antibiotic pressure. The findings will provide an empirical basis for evidence-driven mitigation and conservation strategies tailored to this vulnerable species and its fragile dryland ecosystem. More broadly, this study highlights the gut microbiome—as a functional indicator of environmental pressure and adaptive capacity in wildlife—as a valuable model for assessing species resilience to anthropogenic pollutants on the Qinghai–Tibet Plateau and offers a methodological reference for the conservation of other endangered species inhabiting extreme environments.

## 2. Materials and Methods

### 2.1. Sample Collection

**Figure 1:**
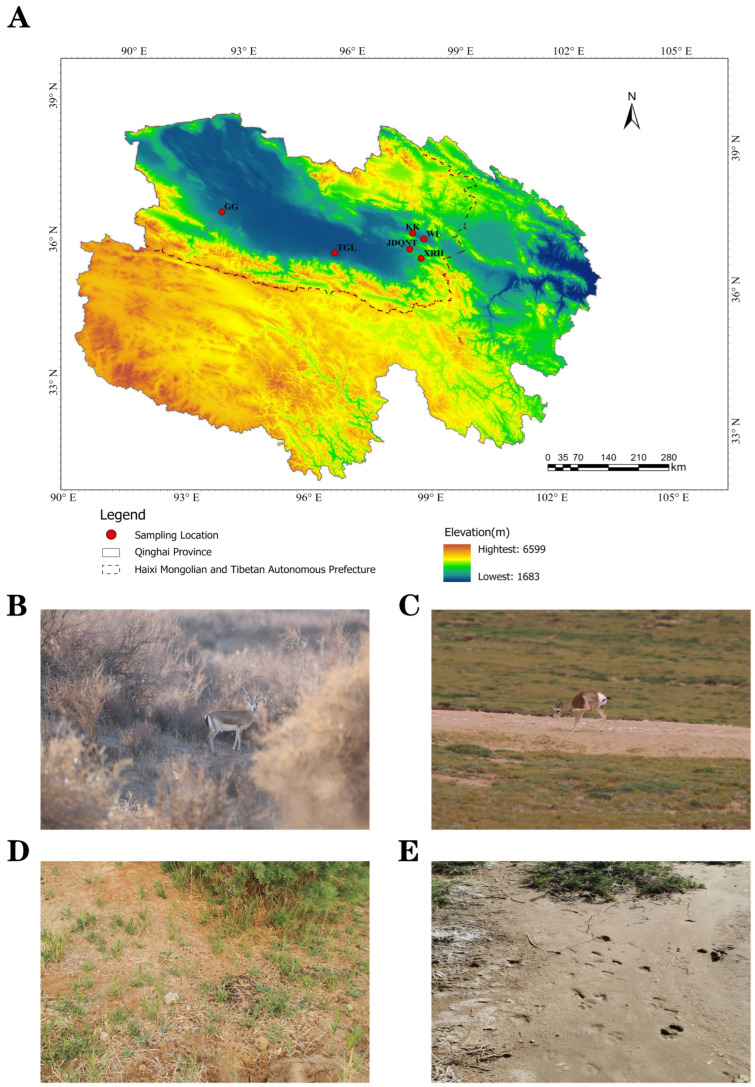
(**A**) Sampling location in the Qaidam Basin, Qinghai province, China (KK: Keke; WL: WuLan; XRH: XiaRiHa; GG: GeGenanmuga; JDQNT: JiaDaQuNiuTeng; and TGL:TianGeLe); (**B**,**C**) the pictures, (**D**) fresh feces, and (**E**) footprints of goitered gazelles.

Based on the spatial distribution of goitered gazelles (*Gazella subgutturosa*) in the Qaidam Basin of Qinghai Province, six sampling sites were selected between 6–21 July 2023: KK (Keke), WL (Wulan), XRH (XiaRiHa), GG (GeGenanmuga), JDQNT (JiaDaQuNiuTeng), and TGL (TianGeLe). At each site, six fresh fecal samples and six adjacent topsoil samples were collected, yielding a total of 72 samples (Figure 1).

All six samples per site were collected within a single day, and no repeated sampling occurred at the same location. Fecal pellets were selected to minimize soil contamination; only the upper surface of each pellet was sampled using disposable polyethylene (PE) gloves, which were replaced after each use. Each fecal sample was placed in a zip-lock bag, labeled, and immediately preserved in liquid nitrogen.

Simultaneously, surface soil samples were collected from the same locations. Soil was sieved through a 60-mesh screen to remove gravel and organic debris, transferred into 5 mL cryotubes, labeled, and immediately frozen in liquid nitrogen. All samples were temporarily stored in liquid nitrogen in the field (for no more than 2 weeks) and subsequently transferred to −80 °C within 1 week prior to DNA extraction.

### 2.2. DNA Extraction, Amplification, and Sequencing

Total genomic DNA was extracted from fecal samples using the E.Z.N.A.^®^ Soil DNA Kit (Omega Bio-tek, Norcross, GA, USA) following the manufacturer’s instructions. DNA quality was verified by 1% agarose gel electrophoresis. The hypervariable V3–V4 region of the bacterial 16S rRNA gene was amplified in triplicate using primers 338F and 806R (5′-ACTCCTACGGGAGGCAGCAG-3′/5′-ACTACHVGGGTWTCTAAT-3′) [19,20]. Polymerase chain reaction (PCR) was performed on an ABI GeneAmp^®^ 9700 thermal cycler (Applied Biosystems, Foster City, CA, USA) under the following conditions: initial denaturation at 95 °C for 3 min, followed by 27 cycles of 95 °C for 30 s, 55 °C for 30 s, and 72 °C for 45 s, with a final extension at 72 °C for 10 min.

Amplicons from triplicate reactions were pooled and purified using a 2% agarose gel and recovered with the AxyPrep DNA Gel Extraction Kit (Axygen Biosciences, Union City, CA, USA). The purified PCR products were quantified using the QuantiFluor™-ST Blue Fluorescence Quantification System (Promega, Madison, WI, USA). Sequencing libraries were prepared at equimolar concentrations (~300 bp insert size) and subjected to paired-end sequencing on the Illumina MiSeq platform (Illumina, San Diego, CA, USA), generating 5,599,762 raw reads. Library preparation and high-throughput sequencing were conducted by Majorbio Bio-Pharm Technology Co., Ltd. (Shanghai, China).

### 2.3. ASV Determination and Taxonomic Classification

ASV determination and taxonomic classification were performed by Majorbio Bio-Pharm Technology, Shanghai, China. Following demultiplexing of paired-end (PE) reads, raw sequences were quality-filtered using Fastp (v0.19.6) to trim low-quality bases (Q-score < 20) [21]. Filtered reads were then merged using FLASH (v1.2.7) with a minimum overlap of 10 bp [22] to generate optimized contigs. To correct residual PCR and sequencing errors, denoising was performed using the DADA2 plugin in QIIME2 [23,24], which removed chimeric sequences, corrected substitution errors, and resolved amplicon sequence variants (ASVs) at single-nucleotide resolution. This process yielded representative ASV sequences and corresponding abundance tables.

Taxonomic classification [25] was conducted using a Naive Bayes classifier implemented in the QIIME2 q2-feature-classifier plugin [26] against the SILVA 138 16S rRNA reference database [27] (confidence threshold = 0.7). ASVs assigned to mitochondria, chloroplasts, or Archaea were excluded from the dataset. Low-abundance ASVs (<1% relative abundance in ≥3 samples) were also removed to reduce noise. All samples were rarefied to a uniform sequencing depth of 54,661 reads per sample prior to downstream comparative analyses.

### 2.4. Antibiotic Concentration Analysis

Fecal and soil samples were analyzed for targeted metabolomics by Shanghai Bioprofile Technology Co., Ltd. (Shanghai, China). Lyophilized fecal samples (0.5 g) or air-dried soil samples (1.0 g) were homogenized with 10 mL of extraction solvent in 15 mL centrifuge tubes. The mixtures were vortexed, sonicated in an ice bath for 20 min, and incubated at 4 °C for 2 h, followed by centrifugation at 7000× *g* for 20 min at 4 °C.

Supernatants (1 mL) were loaded onto pre-conditioned Oasis HLB solid-phase extraction cartridges (1 cc/30 mg, Waters, Milford, MA, USA), activated sequentially with 1 mL methanol and 1 mL ultrapure water. After washing with 1 mL ultrapure water, antibiotics were eluted with 1 mL methanol. The eluates were dried under nitrogen gas and reconstituted in 100 μL of ice-cold 10% methanol. Following centrifugation at 20,000× *g* for 15 min at 4 °C, 50 μL of supernatant was transferred to autosampler vials for LC-MS/MS analysis [28].

Chromatographic separation was performed on a Shimadzu Nexera X2 LC-30AD system (Shimadzu Corporation, Kyoto, Japan) equipped with a Waters ACQUITY UPLC BEH C18 column (2.1 × 100 mm, 1.7 μm, Waters Milford, MA, USA). The mobile phases consisted of 0.1% formic acid in water (A) and 0.1% formic acid in acetonitrile (B). The gradient elution program was as follows: 0–1.5 min, 5% B; 1.5–10 min, 5–55% B (linear); 10–11 min, 55–99% B (linear); 11–13 min, 99% B; 13–13.1 min, 99–5% B (linear); and 13.1–15.1 min, 5% B. The column temperature and flow rate were maintained at 40 °C and 300 μL/min, respectively. Injection volume was 10 μL.

Mass spectrometry was conducted using an AB Sciex 5500 QTrap mass spectrometer (AB SCIEX, Framingham, MA, USA) in electrospray ionization (ESI) positive mode with multiple reaction monitoring (MRM). Data acquisition and peak integration (retention time and peak area) were performed using the MultiQuant software (v3.0.3, AB SCIEX). Quality control (QC) samples—prepared by mixing equal volumes of all test samples—underwent the full pretreatment and detection process alongside actual samples. The relative standard deviation (RSD) of target antibiotic peak areas in QC samples was ≤30%, confirming acceptable repeatability. The external standard method was used for quantification during the determination. The corresponding standard curves were established with 13 standard solutions with concentrations of 0.001, 0.002, 0.005, 0.01, 0.02, 0.05, 0.1, 0.2, 0.5, 1, 2, 5, and 10 µg/mL. Analyte concentrations were calculated by linear regression of the calibration curves (R^2^ > 0.99) and normalized to the sample dry weight.

### 2.5. Statistical and Network Analyses

α-Diversity indices (Shannon and Chao1) were calculated using Mothur software v1.30.2 (https://mothur.org/wiki/calculators/, accessed on 9 October 2024). The Kruskal–Wallis test was applied to assess differences in microbial diversity between groups at a 95% confidence level. False discovery rate (FDR) correction was performed for multiple comparisons [29], and Dunn’s test (α = 0.05) was used for post hoc analysis. β-diversity was assessed based on Bray–Curtis distances using non-metric multidimensional scaling (NMDS), analysis of similarity (ANOSIM), and permutational multivariate analysis of variance (PERMANOVA; 999 permutations) [30], and analysis of multivariate dispersion (betadisper; 999 permutations) to check homogeneity of group dispersions, all implemented via the vegan v2.4.3 package [31]. Visualizations were generated using ggplot2 v3.5.1 [32].

Antibiotic residue concentrations were quantified using Microsoft Excel 2021 (Microsoft Corp., Redmond, WA, USA) and SPSS v27.0 (IBM Corp., Armonk, NY, USA). To evaluate spatial variation, the Kruskal–Wallis test was used to assess differences in soil and fecal antibiotic concentrations among the six regions, with statistical significance set at α = 0.05.

Weighted gene co-expression network analysis (WGCNA) was used to cluster ASVs into microbial modules and evaluate their correlations with 23 antibiotics. Co-abundance networks were constructed using the WGCNA v1.73 package in R v4.4.1 [33]. The network type was set to “signed”, and the soft-threshold power was set to 6 to ensure scale-free topology. Additional parameters included a minimum module size of 30, DeepSplit = 3, and a module merging threshold of 0.25 (i.e., modules with eigengene correlations > 0.75 were merged).

Modules with correlation coefficients |r| ≥ 0.75 and *p* < 0.05 were defined as key modules for downstream analysis. For each ASV within key modules, gene significance (GS) was calculated as the Pearson correlation coefficient between ASV abundance and antibiotic concentration, while module membership (MM) represented the correlation between ASV abundance and the corresponding module eigengene. Hub ASVs—those with high intramodular connectivity—were ranked primarily by |MM|, and secondarily by |GS| when |MM| values were identical. The top five ASVs in each module were retained for further analysis.

## 3. Results

### 3.1. Gut Microbial Diversity

#### 3.1.1. Raw Data

After quality control, a total of 1,967,796 high-quality sequences (mean length: 411 bp) were obtained from 36 fecal samples of goitered gazelles. Taxonomic classification identified 3927 ASVs, spanning 12 phyla, 20 classes, 46 orders, 81 families, and 189 genera. Sample coverage indices exceeded 99%, indicating sufficient sequencing depth.

**Figure 2:**
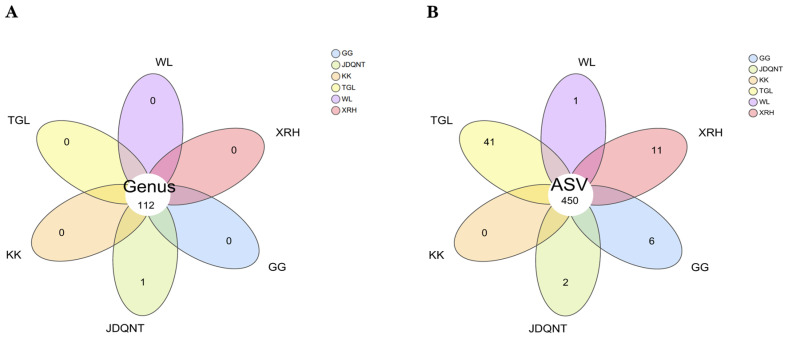
Venn diagram of the gut microbiota of six groups, with taxonomic characterization at (**A**) the genus level and (**B**) the ASV level.

Based on the Venn diagram, at the genus level, 112 genera were shared across all six regions, with the genus *Coprobacillus* being unique to the JDQNT group. At the ASV level, 450 ASVs were shared by the goitered gazelles among the six regions (Figure 2B). The TGL group had the highest number of unique ASVs (*n* = 41), followed by the XRH (*n* = 11) and GG (*n* = 6) groups (Figure 2B).

#### 3.1.2. The Composition of the Gut Microbiota

In the gut microbiota of goitered gazelles, the top five phyla in relative abundance were *Firmicutes*, *Bacteroidota*, *Verrucomicrobiota*, *Actinobacteriota*, and *Patescibacteria*. Among them, *Firmicutes* and *Bacteroidota* dominated the gut microbiota, with mean relative abundances exceeding 67% and 12% in all groups, respectively. Additionally, significant differences in these two phyla were observed among the six groups.

**Figure 3:**
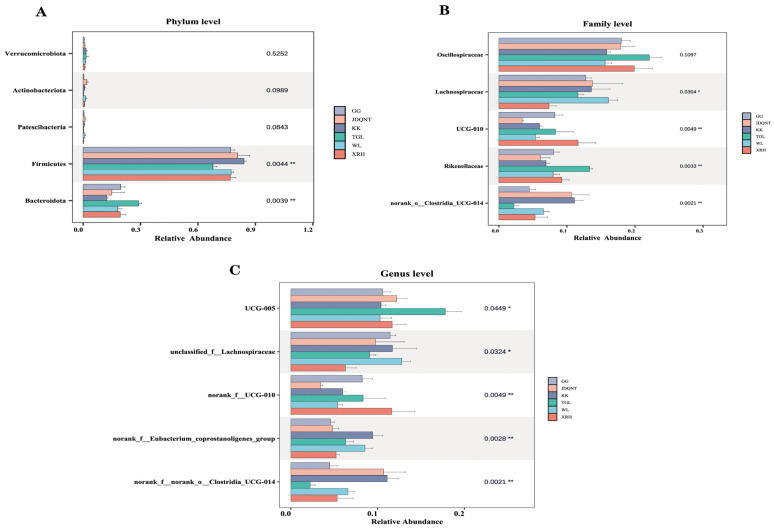
The top five (**A**) phyla, (**B**) families, and (**C**) genera in relative abundance of gut microbiota among six regions based on the Kruskal–Wallis test (F represents fecal; * represents *p* < 0.05; ** represents 0.001 < *p* < 0.01).

At the family level, the top five families were *Oscillospiraceae*, *Lachnospiraceae*, *Rikenellaceae*, *UCG-010*, and *norank_o__Clostridia_UCG-014*, and only *Oscillospiraceae* showed no significant differences among the six groups (*p* > 0.05, Figure 3B). At the genus level, the top five genera were *UCG-005*, *unclassified_f__Lachnospiraceae*, *norank_f__UCG-010*, *norank_f__norank_o__Clostridia_UCG-014*, and *norank_f__Eubacterium_coprostanoligenes_group*, all of which showed significant differences (*p* < 0.05; Figure 3C).

#### 3.1.3. Analysis of Alpha and Beta Diversity

**Figure 4:**
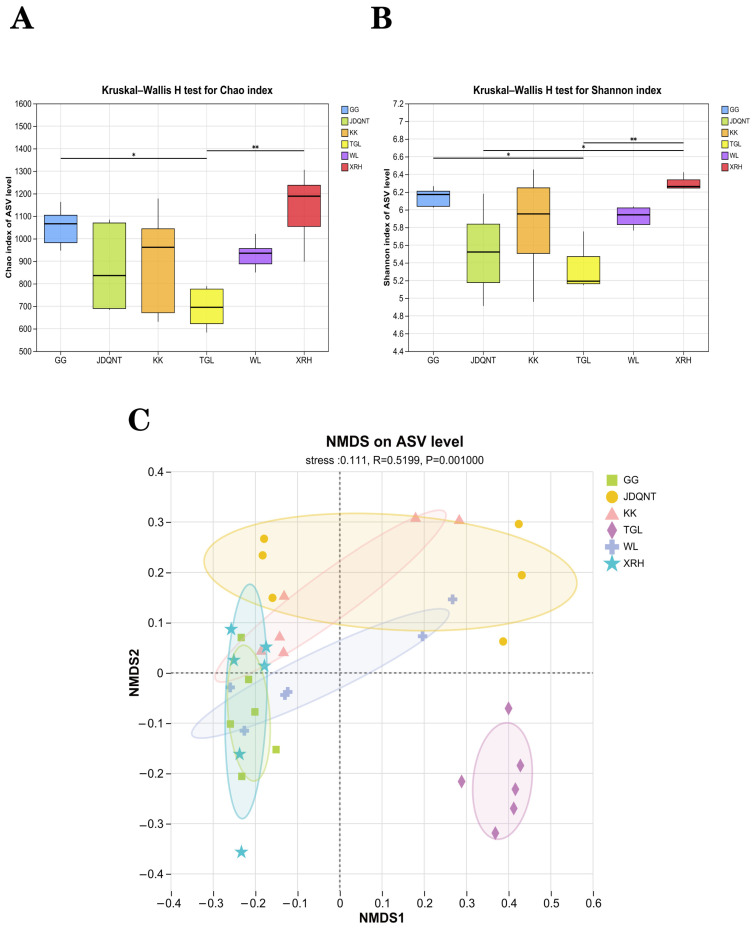
The α-diversity of the (**A**) Chao1 index and (**B**) Shannon index in soil microbiota at the ASV level among six groups based on the Kruskal–Wallis test (F represents fecal; * represents *p* < 0.05; ** represents 0.001 < *p* < 0.01). (**C**) NMDS analysis among six regions of gut microbiota at the ASV level based on Bray–Curtis distance matrices.

The alpha diversity of the six groups showed that at the ASV level, the values of the Shannon (*p* = 0.004 < 0.05, Figure 4A) and Chao1 (*p* = 0.004 < 0.05, Figure 4B) indices in the XRH groups were highest. In contrast, the α-diversity of the gut microbiota in the TGL, evaluated at the ASV level, exhibited the lowest. These differences were statistically significant, as denoted by the criterion of *p* < 0.05, indicating that the gut microbial diversity in the TGL groups was notably impoverished when compared to the other groups.

In terms of β-diversity, the permutation test for homogeneity of multivariate dispersions revealed no statistically significant differences in dispersion among groups (*F* value = 1.612, *p* = 0.187), satisfying the prerequisite for PERMANOVA. Subsequently, PERMANOVA analysis revealed significant differences in gut microbiota diversity among the six groups (R^2^ = 0.292, *p* = 0.001), corroborated by ANOSIM (0.520, *p* = 0.001). NMDS ordination confirmed that inter-regional differences exceeded intra-group variability (stress = 0.111, *p* = 0.001), with the TGL group clearly separated from the other five groups (Figure 4C).

### 3.2. Antibiotics in Soil and Feces

**Figure 5:**
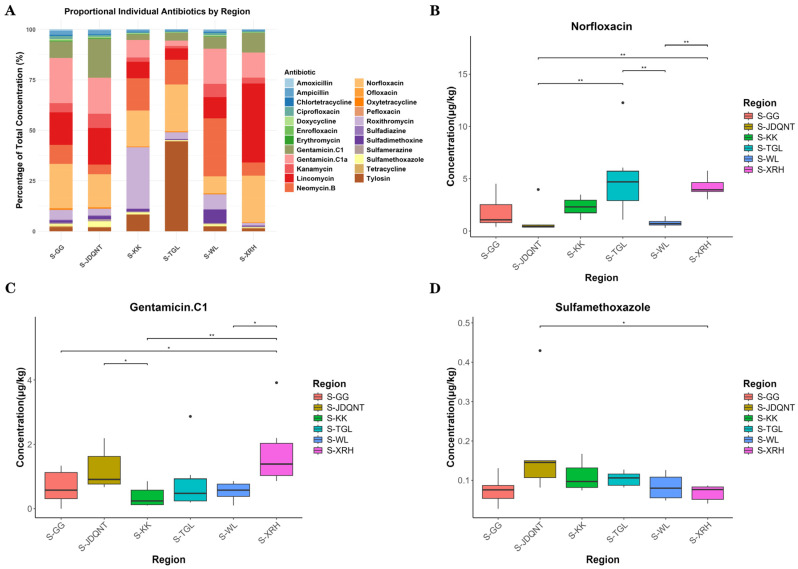
(**A**) Stacked bar chart of antibiotic concentrations in soil among six groups. Boxplots of soil. (**B**) Norfloxacin, (**C**) Gentamicin C1, and (**D**) Sulfamethoxazole residues with significant spatial variations. *p* values for group comparisons were calculated by the nonparametric Kruskal–Wallis test. * *p* < 0.05; ** *p* < 0.01.

Antibiotic concentrations and detection frequencies in 36 soil samples are summarized in Table S1. Among the 23 antibiotics analyzed, detection frequencies ranged from 72.22% to 100%, indicating widespread contamination across sites (Table S1). The proportional distribution of the 23 antibiotics across the six sampling regions is shown in Figure 5A. Kruskal–Wallis tests with Dunn’s post hoc comparisons revealed significant spatial differences in the concentrations of norfloxacin, gentamicin C1, and sulfamethoxazole (*p* < 0.05). Among these, norfloxacin exhibited the highest overall residual concentration (Figure 5B–D). The TGL and XRH groups showed the highest soil antibiotic levels; however, no significant difference was observed between these two groups (*p* > 0.05; Figure 5B).

**Figure 6:**
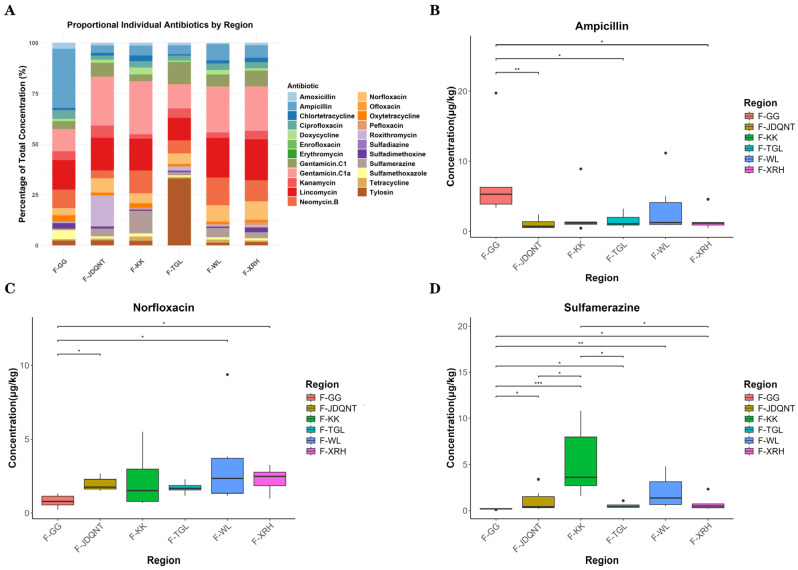
(**A**) Stacked bar chart of antibiotic concentrations in feces among six groups. Boxplots of the top three antibiotics (by overall residual concentration) among the 16 antibiotics with significant spatial differences: (**B**) Ampicillin, (**C**) Norfloxacin, (**D**) Sulfamerazine. *p* values for group comparisons were calculated by the nonparametric Kruskal–Wallis test. * *p* < 0.05; ** *p* < 0.01; *** *p* < 0.001.

Antibiotic concentrations in 36 fecal samples and their detection frequencies are presented in Table S2. Among the 23 antibiotics analyzed, detection frequencies in fecal samples ranged from 83.33% to 100%, indicating widespread antibiotic exposure (Table S2). The proportional distribution of 23 antibiotics in fecal sampling sites among six regions is shown in Figure 6A. Kruskal–Wallis tests revealed no significant spatial differences for eight fecal antibiotics (tylosin, roxithromycin, kanamycin, gentamicin C1, gentamicin C1a, neomycin B, lincomycin, amoxicillin) among the six regions; the remaining 15 antibiotics showed significant spatial variation. Among the 16 antibiotics with significant spatial differences, ampicillin showed the highest overall residual concentration, followed by norfloxacin and sulfamerazine (Figure 6B–D).

### 3.3. Identification of Antibiotic-Associated Core ASVs via WGCNA

**Figure 7:**
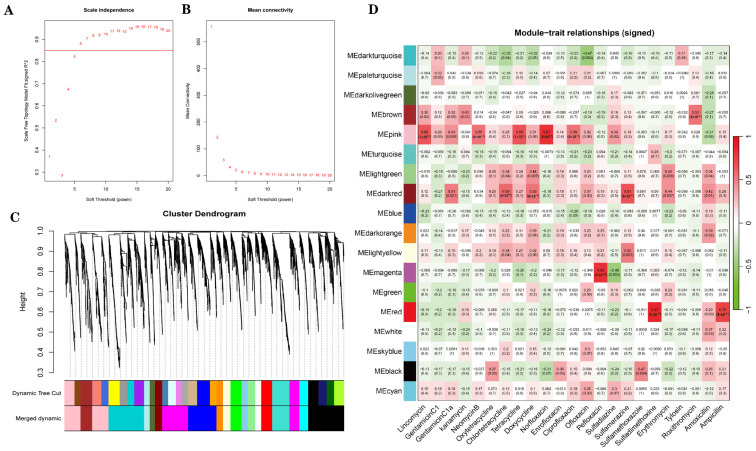
(**A**,**B**) Determination of the soft-thresholding power. (**A**) The *y*-axis represents the scale-free topology fitting index (R^2^) corresponding to different β values. When the soft-thresholding power is set to 6, the R^2^ value exceeds 0.85, indicating an approximate scale-free topology. (**B**) The *y*-axis shows the mean connectivity of nodes under different β values. (**C**) Hierarchical clustering dendrogram of ASVs, with branches corresponding to distinct modules. (**D**) Heatmap showing the correlations between module eigengenes (rows) and antibiotic traits (columns). Each cell indicates the Pearson correlation coefficient and corresponding *p*-value, with red denoting positive correlations and green denoting negative correlations.

As shown in Figure 7A, when soft-thresholding power was set to 6, the scale-free topology fitting index (R^2^) was greater than 0.85. The TOM-based hierarchical clustering was performed, and the dynamic tree cut method was used to identify the co-expression modules. A total of 3927 amplicon sequence variants (ASVs) were assigned to 33 different color modules, which were subsequently merged into 18 modules based on similarity (Figure 7C). According to the correlation between modules and antibiotics, three modules were selected based on |r| ≥ 0.75 and *p* < 0.05. Figure 7D shows module–antibiotic correlations. Among them, the pink module was positively correlated with norfloxacin (r = 0.81, *p* = 2 × 10^−9^), the red module was positively correlated with ampicillin (r = 0.76, *p* = 8 × 10^−8^) and sulfadimethoxine (r = 0.87, *p* = 5 × 10^−12^), the magenta module was positively correlated with pefloxacin (r = 0.83, *p* = 5 × 10^−10^).

**Figure 8:**
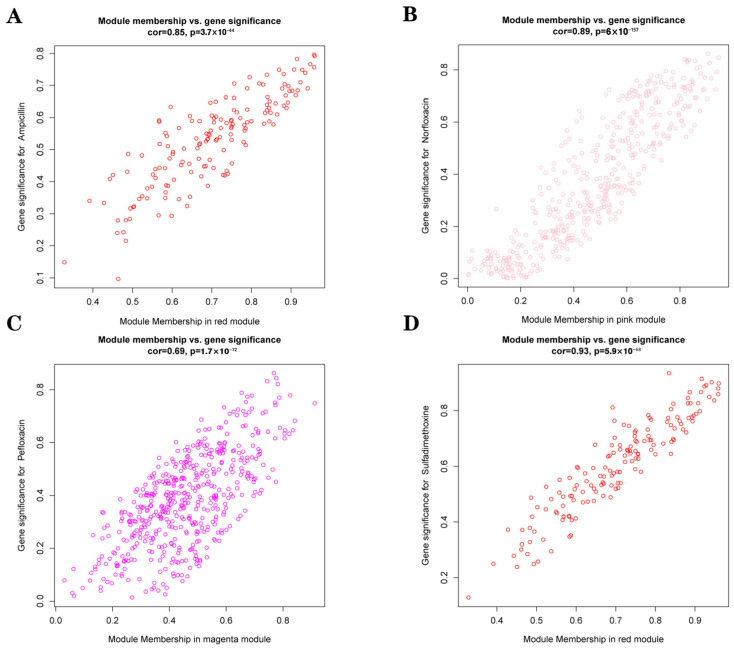
Scatter plot of GS and MM. (**A**) Ampicillin associated with the red module; (**B**) Norfloxacin associated with the pink module; (**C**) Pefloxacin associated with the magenta module; (**D**) Sulfadimethoxine associated with the red module.

We generated scatter plots of gene significance (GS) versus module membership (MM) for each module associated with the corresponding antibiotics (Figure 8). The top five ASVs in each of the three key modules were ranked primarily by the absolute value of MM (|MM|) and secondarily by the absolute value of GS (|GS|) in cases of ties (Table S3). These top ASVs were further identified to the genus level.

In the pink module, *Mogibacterium* (ASV11643), *Eubacterium_nodatum_group* (ASV11744), *Eubacterium_hallii_group* (ASV11670, ASV11980), and *Blautia* (ASV22580) were the core ASVs of this module.

In the red module, *unclassified_c__Clostridia* (ASV24884, ASV1367), *unclassified_f__Lachnspiraceae* (ASV7256), *Candidatus_Saccharimonas* (ASV7368), and *norank_f__norank_o__Clostridia_UCG-014* (ASV7417) were the core ASVs of this module.

In the magenta module, the five core ASVs were as follows: *norank_f__norank_o__Clostridia_vadinBB60_group* (ASV7205), *norank_f__UCG-010* (ASV88), *unclassified_f__Eggerthellaceae* (ASV5179), *unclassified_f__Oscillospiraceae* (ASV5054), and *CAG-352* (ASV7129).

## 4. Discussion

### 4.1. Combined Dietary and Antibiotic Pressures Reduce Gut Microbial α-Diversity

Diet is one of the primary factors influencing the development, structure, and maturation of gut microbiota in herbivores [34,35]. Dietary composition directly affects gut microbial richness and diversity, as it determines the types and availability of substrates that support microbial growth [36]. In our study, α-diversity analyses revealed that the gut microbiota of goitered gazelles in the TGL region exhibited the lowest diversity among all sampled areas. This region is characterized by sparse vegetation dominated by *Tamarix arceuthoides* and *Phragmites communis*, supporting fewer plant species compared to the XRH region, which displayed the highest gut microbial diversity [37]. The lowest diversity of gut microbiota in TGL is likely attributable to limited plant diversity and nutritional resource constraints [38].

In addition to diet, environmental antibiotic residues play a critical role in shaping gut microbial communities by altering both microbial composition and abundance [39]. Antibiotic exposure can suppress gut microbial diversity, leading to dysbiosis and reduced microbial resilience [40]. Among the 23 antibiotics in both soil and fecal concentration across six sites, norfloxacin was relatively high in the TGL region (Figure 5B and Figure 6C). We propose that the low gut microbial diversity observed in TGL is the result of synergistic pressures from both limited dietary plant diversity and high antibiotic exposure.

This dual pressure may lead to the gut microbiota of goitered gazelles in the TGL that is simplified yet energetically efficient—potentially an adaptive strategy in resource-poor environments. The gut microbial community may reduce the metabolic cost associated with maintaining complex microbial networks, allowing the goitered gazelles to conserve energy [41]. However, this energetic trade-off comes at a cost: as microbial diversity and functional redundancy decline, the goitered gazelles in the TGL become more vulnerable to metabolic, immune, and inflammatory dysregulation, potentially compromising overall physiological homeostasis [42].

Wang et al. previously reported that wild goitered gazelles inhabiting harsher environments show higher dependence on environmental soil microbiota [43]. This implies that goitered gazelles in ecologically stressed regions like TGL may engage more frequently in soil-licking behavior as a compensatory strategy to acquire exogenous microbes or micronutrients. However, such behavior also increases the likelihood of ingesting soil-borne antibiotic residues. Under these conditions, the goitered gazelles’ gut microbiota may be subjected to continued antibiotic stress in the absence of sufficient dietary inputs [44]. As a result, in harsh environments, the gut microbiota of goitered gazelles may experience loss of diversity and sensitivity to antibiotic pressure, potentially leading to the emergence of a region-specific but fragile gut microbial community structure.

### 4.2. High Vegetation Diversity Buffers Antibiotic Pressure

By contrast, gut microbial α-diversity was highest in the XRH region. The XRH region is characterized by high plant species richness, dominated by diverse semi-arid and desert shrub communities (e.g., *Kalidium foliatum*, *Nitraria tangutorum*, and allied species) [37], possibly providing goitered gazelles with a markedly more diverse diet than in TGL. Populations subsisting on heterogeneous forage generally sustain richer gut microbial diversity [45]. For example, Brandt’s voles show a monotonic increase in gut microbial α-diversity with greater plant-species richness [46]. We therefore infer that XRH’s high vegetation diversity and the associated ecological conditions contribute to the significantly higher α-diversity observed in XRH compared to TGL. This indicates a mechanism distinct from the combined vegetation–antibiotic pressure proposed for XRH, when TGL and XRH face the same high pressure of norfloxacin (no significant difference is shown in the concentration of norfloxacin in either soil or feces between XRH and TGL): when dietary diversity is high, antibiotic pressure alone does not appear to suppress gut microbial richness.

The increase in vegetation diversity and α-diversity of gut microbiota confers ecological robustness—greater resistance to disturbance, faster homeostatic recovery, and enhanced adaptive capacity [47]. In ruminants, fiber-rich, low-fat diets strengthen symbiotic networks and accelerate restitution from antibiotic-induced dysbiosis [48]. We therefore propose that XRH’s heterogeneous vegetation base supports a functionally versatile microbiome capable of sustaining metabolic performance and mitigating norfloxacin toxicity despite high environmental antibiotic exposure.

### 4.3. Key Antibiotics Impact Gut Microbiota via Core ASV Modules

The gut microbiota typically exists in a state of dynamic equilibrium; however, external disturbances such as antibiotic exposure or pathogenic infection can disrupt its structure and functional stability [49]. Different classes of antibiotics elicit distinct microbial responses in hosts, depending on their pharmacodynamic targets and metabolic characteristics. These responses often manifest as shifts in the abundance of specific microbial taxa [50]. In the present study, weighted gene co-expression network analysis (WGCNA) identified four antibiotics—pefloxacin, norfloxacin, ampicillin, and sulfadimethoxine—as primary environmental stressors (|r| ≥ 0.75, *p* < 0.05) that exhibited the strongest correlations with major gut microbial modules (specifically the ‘pink’, ‘magenta’, and ‘red’ modules) in goitered gazelles.

The data support the conclusion that these antibiotics play a dominant role in modulating gut microbial community composition. To further explore the ecological implications of these modules, we investigated their internal composition to identify bacterial taxa that may serve as hub ASVs (amplicon sequence variants). Hub ASVs are central nodes in microbial co-occurrence networks that may exert outsized influence on community stability and functional traits, especially under environmental stress. By characterizing these hub taxa, we aimed to better understand how specific microbial lineages mediate module-level responses to antibiotic exposure and contribute to microbiome restructuring in the context of antibiotic-contaminated environments [51].

#### 4.3.1. Gut Microbial Buffering and Risk Under Norfloxacin Pressure

The “pink” module displayed the strongest positive correlation with norfloxacin, a broad-spectrum fluoroquinolone that rapidly inhibits bacterial DNA replication and repair in a wide range of Gram-negative and selected Gram-positive taxa [52]. Gene significance (GS) and module membership (MM) analyses identified four dominant genera within this module—*Eubacterium_hallii_group*, *Mogibacterium*, *Blautia*, and *Eubacterium_nodatum_group*.

The genera *Blautia* [53], *Eubacterium_hallii_group* [54], and *Eubacterium_nodatum_group* [55] are well-known producers of short-chain fatty acids (SCFAs) such as acetate and butyrate. SCFAs serve dual roles: (i) they supply metabolic energy to both host and microbiota, and (ii) they regulate mucosal immunity while reinforcing intestinal-barrier integrity in goitered gazelles [56]. In addition, *Mogibacterium* can assimilate excess ammonia, thereby mitigating luminal ammonia toxicity [57]. Collectively, these functions constitute a metabolic buffering network in the gut microbiome of goitered gazelles under norfloxacin stress, supporting energy production, epithelial barrier integrity, and nitrogenous waste detoxification, thereby enhancing tolerance to antibiotic exposure.

However, enrichment of these potentially antibiotic-resistant taxa also carries health risks in goitered gazelles [58]. Elevated abundances of the *E. hallii group* could potentially disrupt enterohepatic bile acid circulation (this remains a hypothesis that warrants further investigation), which might then impair lipid emulsification and absorption and ultimately predispose adiposity and skeletal muscle deficits [59]. In wild goitered gazelles, such physiological disturbances may impair locomotor performance, lower foraging efficiency, disrupt metabolic homeostasis, and compromise energy storage essential for winter survival. Overgrowth of the *E. nodatum group* can increase levels of secondary bile acids such as deoxycholic acid and cholic acid, metabolites linked to colorectal carcinogenesis [60]. Emerging evidence links *Mogibacterium* overabundance with reduced pulmonary function in other species. Although direct validation in goitered gazelles is currently unavailable, this association presumes a potential gut–lung axis mechanism that may adversely affect host physiological resilience [61].

Thus, while the pink-module taxa appear to form a functional shield against norfloxacin toxicity in goitered gazelles, their proliferation simultaneously heightens metabolic and inflammatory risks for the host—illustrating the trade-offs inherent in microbiome-mediated adaptation to antibiotic-contaminated environments.

#### 4.3.2. Trade-Offs in Gut Microbial Remodeling Under Pefloxacin Pressure

The magenta module showed a strong positive association with pefloxacin, a broad-spectrum fluoroquinolone. Gene-significance and module-membership analyses identified one dominant genus—*CAG-352*—together with three core families: *Ruminococcaceae*, *Eggerthellaceae*, and *Oscillospiraceae*.

Under pefloxacin stress, enrichment of *CAG-352*, *Ruminococcaceae*, and *Eggerthellaceae* appears to build a synergistic metabolic network in goitered gazelles. *CAG-352* and bacteria of *Ruminococcaceae* attain high relative abundance, likely because they enhance degradation of complex plant fibers (pectin, cellulose) and may help stabilize intestinal permeability [62,63]. *Eggerthellaceae* improves feed efficiency through flavonoid metabolism and additional fiber digestion—traits that are especially valuable in the nutrient-poor diets typical of arid grasslands [64].

For wild goitered gazelles, these shifts supply readily utilizable energy and micronutrients, creating a short-term buffering effect against antibiotic toxicity. However, the restructuring carries metabolic costs: Overgrowth of *Oscillospiraceae* can erode the mucin scaffold of the gut barrier, predisposing animals to systemic inflammatory responses [65]. The *CAG-352* group has been linked to the pathogenesis of pulmonary tuberculosis, raising concerns about potential gut–lung-axis repercussions in goitered gazelles [66].

In summary, pefloxacin exposure promotes gut microbial functions that improve fiber breakdown and flavonoid utilization, offering immediate survival benefits to goitered gazelles. Yet, the concurrent enrichment of pro-inflammatory and potentially pathogenic taxa may undermine long-term resilience to additional stresses such as drought or parasitism.

#### 4.3.3. Gut Microbial Trade-Offs Under Ampicillin/Sulfadimethoxine Pressure

The red module was strongly associated with combined exposure to ampicillin and sulfadimethoxine. Within this cluster, *Candidatus Saccharimonas* displayed the highest positive loading, indicating that its proliferation is selectively favored under β-lactam and sulfonamide pressure. Previous studies indicate that Ca. *Saccharimonas* exerts immunomodulatory effects: its abundance is inversely related to hepatic injury indices, pro-inflammatory cytokines, circulating lipopolysaccharide, and JNK phosphorylation, while correlating positively with antioxidant markers such as the Bcl-2/Bax ratio and superoxide dismutase activity [67,68]. These properties imply a protective role that could reinforce epithelial-barrier integrity and limit pathogen translocation in goitered gazelles experiencing antibiotic stress.

However, enrichment of Ca. *Saccharimonas* also carries metabolic trade-offs. The taxon thrives under highly fermentable diets and is linked to ruminal pH depression; the ensuing acidification promotes lysis of Gram-negative bacteria, elevating luminal LPS and lactate loads [69]. Consequently, while ampicillin/sulfadimethoxine-induced proliferation of Ca. *Saccharimonas* may enhance antioxidant defenses, it may simultaneously predispose goitered gazelles to endotoxemia and acidosis—highlighting the dualistic nature of microbiome-mediated adaptation to antibiotic exposure.

Exposure to key antibiotics—including norfloxacin, pefloxacin, ampicillin, and sulfadimethoxine—profoundly reshaped the gut microbiota of goitered gazelles through the activation of core microbial modules. While enriched bacteria supported metabolic buffering via SCFA production, flavonoid metabolism, and ammonia detoxification, they also introduced physiological risks such as impaired bile acid homeostasis, mucosal inflammation, and systemic toxicity. These findings highlight the dualistic nature of microbiome-mediated responses under antibiotic pressure and emphasize the ecological trade-offs between short-term resilience and long-term host vulnerability.

## 5. Conclusions

Environmental antibiotic pressure—particularly norfloxacin, pefloxacin, and ampicillin/sulfadimethoxine—plays a dominant role in restructuring the gut microbiota of wild goitered gazelles in the Qaidam Basin. Antibiotic exposure was significantly associated with modular shifts in microbial composition, including enrichment of SCFA-producing and antioxidant-associated taxa. These shifts may offer short-term metabolic buffering but also elevate host risks via bile acid disruption, mucosal barrier damage, and systemic inflammation.

While vegetation diversity may provide partial resilience, antibiotic pressure alone was sufficient to restructure microbial communities. Gut microbial α-diversity was lowest in the vegetation-sparse, antibiotic-rich TGL region, consistent with the compounded effects of resource limitation and antibiotic stress. In contrast, high vegetation diversity in the XRH region was associated with greater gut microbial richness, potentially buffering antibiotic impact.

Overall, our findings highlight the dual role of gut microbiota in mediating resilience and risk under antibiotic pressure and underscore the need for habitat-level monitoring of antimicrobial contamination in arid ecosystems. Goitered gazelles may serve as effective sentinels for assessing such ecological impacts.

We consider that conserving and restoring native vegetation—particularly *Ceratoides*, *Artemisia*, and *Krascheninnikovia* spp.—within goitered gazelle habitats may serve as an effective ecological buffer against environmental antibiotic stress. By promoting plant diversity and dietary heterogeneity, such habitats can enhance gut microbiota resilience, reduce microbial dysbiosis, and mitigate the influence of antibiotic-resistant taxa. This strategy offers a feasible and nature-based intervention for antibiotic risk management in the Qaidam Basin, complementing broader habitat conservation efforts without requiring intensive artificial measures.

To mitigate microbiome-mediated physiological trade-offs associated with antibiotic exposure, we recommend establishing livestock exclusion zones and implementing rotational grazing strategies near ecologically sensitive habitats of goitered gazelles. To evaluate intervention effectiveness and track ecological risk, we further propose a long-term monitoring framework incorporating (i) spatiotemporal profiling of key antibiotic residues—particularly norfloxacin, pefloxacin, ampicillin, and sulfadimethoxine—in soil and water matrices and (ii) targeted surveillance of core microbial modules (e.g., ‘pink’, ‘magenta’, ‘red’) and their indicator taxa (e.g., *Mogibacterium*, *CAG-352*, *Candidatus Saccharimonas*) in fecal samples. Together, these strategies provide a practical and evidence-based approach for sustaining gut microbial resilience and host health in antibiotic-impacted arid ecosystems.

## Data Availability

The Illumina sequencing data used in this study have been submitted to the National Genomics Data Center (NGDC) of China https://ngdc.cncb.ac.cn (accessed on 26 June 2025), bioproject: PRJCA034754 and bioproject: PRJCA042246.

## Supplementary Materials

The following supporting information can be downloaded at: https://www.mdpi.com/article/10.3390/microorganisms13081842/s1. Table S1. Descriptive statistics for the residue levels of 23 kinds of antibiotics in soil samples (μg·kg^−1^). Table S2. Descriptive statistics for the residue levels of 23 kinds of antibiotics in fecal samples (μg·kg^−1^). Table S3. The top five selected ASVs, based on GS and MM values.

## Abbreviations

The following abbreviations are used in this manuscript:

CAZy	Carbohydrate-active enzyme
LC-MS/MS	Liquid chromatography tandem mass spectrometry
ANOSIM	Analysis of similarities
NMDS	Nonmetric multidimensional scaling
PERMANOVA	Permutational multivariate analysis of variance
