# Supplementary material for: The Gut Microbial Adaptation of Wild Goitered Gazelles Under Antibiotic Pressure in the Qaidam Basin
Source: Microorganisms. 2025 Aug 7;13(8):1842. doi: 10.3390/microorganisms13081842 (PMC12388149; doi:10.3390/microorganisms13081842)
Supplement: Supplementary file 1 [file microorganisms-13-01842-s001.zip › microorganisms-3779866-supplementary.pdf]

Table S1. Descriptive statistics of the residue levels of 23 antibiotics in soil samples (μg·kg^{-1}). Red markings indicate antibiotics whose concentrations showed significant spatial differences among the six sampling regions (Kruskal–Wallis test, $p < 0.05$).

Antibiotic class	Substance	Sample size	Occurrence (%)	Geometric mean	Minimum	Maximum	Standard deviation
β-lactam	Ampicillin	36	100.00	0.12	0.08	0.16	0.03
MLs	Erythromycin	36	100.00	0.01	0.01	0.02	0.00
	Tylosin	36	97.22	1.97	0.12	9.98	3.94
	Roxithromycin	36	100.00	1.02	0.19	3.95	1.45
AGs	Kanamycin	36	91.67	0.42	0.26	0.60	0.14
	Gentamicin C1	36	97.22	0.91	0.37	1.78	0.52
	Gentamicin C1a	36	100.00	1.42	0.58	2.26	0.60
	Neomycin B	36	72.22	1.62	0.30	2.77	1.02
TCs	Oxytetracycline	36	91.67	0.03	0.02	0.05	0.01
	Chlortetracycline	36	100.00	0.03	0.02	0.04	0.01
	Tetracycline	36	100.00	0.03	0.01	0.04	0.01
	Doxycycline	36	100.00	0.04	0.03	0.05	0.01
LDs	Lincomycin	36	100.00	2.15	0.96	7.13	2.45
QNs	Norfloxacin	36	100.00	2.55	0.77	5.19	1.78
	Pefloxacin	36	100.00	0.03	0.01	0.07	0.02
	Enrofloxacin	36	100.00	0.02	0.02	0.04	0.01
	Ciprofloxacin	36	100.00	0.09	0.06	0.13	0.03
	Ofloxacin	36	100.00	0.02	0.01	0.02	0.00
Sas	Sulfadiazine	36	100.00	0.02	0.02	0.03	0.00
	Sulfamerazine	36	100.00	0.05	0.04	0.07	0.01
	Sulfamethoxazole	36	100.00	0.10	0.07	0.17	0.04
	Sulfadimethoxine	36	100.00	0.19	0.07	0.61	0.21
PCNs	Amoxicillin	36	97.22	0.04	0.02	0.06	0.01

Table S2. Descriptive statistics of the residue levels of 23 antibiotics in fecal samples (μg·kg^{-1}). Red markings indicate antibiotics whose concentrations showed significant spatial differences among the six sampling regions (Kruskal–Wallis test, $p < 0.05$).

Antibiotic class	Substance	Sample size	Occurrence (%)	Geometric mean	Minimum	Maximum	Standard deviation
β-lactam	Ampicillin	36	100.00	2.86	1.06	7.28	2.32
MLs	Erythromycin	36	100.00	0.02	0.02	0.04	0.01
	Tylosin	36	100.00	2.38	0.42	11.04	4.25
	Roxithromycin	35	100.00	0..97	0.10	34.00	13.56
AGs	Kanamycin	36	97.22	1.26	1.03	1.66	0.27
	Gentamicin C1	36	91.67	2.00	0.83	3.60	0.95
	Gentamicin C1a	36	100.00	6.86	2.73	12.37	3.62
	Neomycin B	36	83.33	3.22	1.07	5.90	1.93
TCs	Oxytetracycline	36	100.00	0.48	0.22	0.95	0.28
	Chlortetracycline	36	100.00	0.57	0.23	1.31	0.40
	Tetracycline	36	100.00	0.42	0.17	1.02	0.34
	Doxycycline	36	100.00	0.61	0.21	1.56	0.54
LDs	Lincomycin	36	100.00	5.48	3.63	8.40	1.99
QNs	Norfloxacin	36	100.00	2.05	0.81	3.39	0.84
	Pefloxacin	36	100.00	0.16	0.07	0.38	0.12
	Enrofloxacin	36	100.00	0.13	0.09	0.21	0.05
	Ciprofloxacin	36	100.00	1.01	0.54	1.50	0.37
	Ofloxacin	36	100.00	0.14	0.08	0.22	0.06
SAs	Sulfadiazine	36	100.00	0.10	0.06	0.14	0.03
	Sulfamerazine	36	100.00	1.64	0.18	5.24	1.87
	Sulfamethoxazole	36	100.00	0.54	0.28	1.14	0.35
	Sulfadimethoxine	36	100.00	0.34	0.15	0.64	0.21
PCNs	Amoxicillin	36	100.00	0.44	0.27	0.72	0.19

Table S3. The top 5 ASVs selected based on GS and MM values.

probes	moduleColor	MM	GS	absMM	absGS
ASV7205	magenta	0.911491	-0.09844	0.911491	0.098443
ASV88	magenta	0.841655	-0.07728	0.841655	0.077284
ASV5179	magenta	0.835303	-0.07189	0.835303	0.071889
ASV5054	magenta	0.825456	0.026376	0.825456	0.026376
ASV7129	magenta	0.822315	0.008449	0.822315	0.008449
ASV11643	pink	0.945703	0.660004	0.945703	0.660004
ASV11744	pink	0.938311	0.719205	0.938311	0.719205
ASV11670	pink	0.93418	0.59712	0.93418	0.59712
ASV22580	pink	0.917494	0.646576	0.917494	0.646576
ASV11980	pink	0.911436	0.752179	0.911436	0.752179
ASV24884	red	0.959764	-0.13029	0.959764	0.130289
ASV7256	red	0.958477	-0.14198	0.958477	0.141978
ASV7368	red	0.95836	-0.19231	0.95836	0.192308
ASV7417	red	0.948578	-0.20678	0.948578	0.20678
ASV1367	red	0.942071	-0.10567	0.942071	0.10567
